# A Video Protocol of Retroviral Infection in Primary Intestinal Organoid Culture

**DOI:** 10.3791/51765

**Published:** 2014-08-11

**Authors:** Amanda Andersson-Rolf, Juergen Fink, Roxana C. Mustata, Bon-Kyoung Koo

**Affiliations:** ^1^Department of Genetics, University of Cambridge; ^2^Wellcome Trust - Medical Research Council Stem Cell Institute, University of Cambridge

**Keywords:** Genetics, Issue 90, Retrovirus, Lentivirus, Organoid culture, Lgr5, Intestine, 3Rs

## Abstract

Lgr5-positive stem cells can be supplemented with the essential growth factors Egf, Noggin, and R-Spondin, which allows us to culture ever-expanding primary 3D epithelial structures *in vitro*. Both the architecture and physiological properties of these 'mini-guts', also called organoids, closely resemble their *in vivo* counterparts. This makes them an attractive model system for the small intestinal epithelium. Using retroviral transduction, functional genetics can now be performed by conditional gene overexpression or knockdown. This video demonstrates the procedure of organoid culture, the generation of retroviruses, and the retroviral transduction of organoids to assist phenotypic analysis of the small intestinal epithelium *in vitro*. This novel organotypic model system in combination with retroviral mediated gene expression provides a valuable tool for rapid analysis of gene function *in vitro* without the need of costly and time-consuming generation for transgenic animals.

**Figure:**
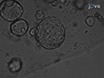


## Introduction

High-throughput functional genetics is needed to increase our biological understanding of the body, to improve current basic science and medicine. Mouse genetics has been the gold standard for investigating gene function* in vivo*, although it is both time-consuming and costly. Cell lines, being the other common choice, have a higher throughput capacity while being less expensive. However, they are impeded by their inability to reproduce the proper microenvironment and thereby the physiological responses seen *in vivo.* Hence, there is an unambiguous need for an easy-to-handle model system, which allows cost/time-efficient high throughput analysis while mimicking the physiological responses observed in *in vivo* transgenic (tg) mouse experiments.

For the endodermal epithelium one such model system appeared in 2009^1^. Among the knowledge gained from the discovery of Lgr5-positive intestinal stem cells was information about the niche corresponding to the extra-cellular matrix and growth factors necessary for stem cell maintenance. Utilizing this information it became possible to establish ‘mini-guts’ also known as organoids^2^. Recently a consensus nomenclature for* in vitro* cultures, where organoids are referred to as ‘enteroids’, was suggested^3^. Like cell lines, the organoids are ever-expanding and easy to treat with ligands and inhibitors. However, instead of being two-dimensional they are three-dimensional self-organizing structures that retain the crypt-villus organization as well as stem cells and differentiated cell lineages of the small intestine (SI). Organoids consist of a single layer of epithelial cells that surround a luminal area. Protruding budding structures correspond to small intestinal crypts containing the stem cell compartment. Starting from the tip of the budding structure progenitor cells differentiate as they migrate towards the epithelial lining, where terminally differentiated cells are shed into the lumen. Compared to cell lines, this *ex vivo* system more closely recapitulates the normal physiology and is therefore a promising model system for the small intestinal epithelium.

In this video protocol of retroviral transduction, we present a method that enables *ex vivo *gene function studies in this novel organoid culture system. We start by describing organoid culture in a step-by-step manner, and continue by demonstrating the generation of retroviruses followed by the transduction procedure. Finally, there is a section for additional advice for troubleshooting. An advantage of this technique is that it can be combined with live imaging or drug screening to study homeostasis, cell fate decisions and cell-cell interactions in the intestinal epithelium. Due to their simple architecture and fast turnover rate, organoids represent an ideal model system for studying adult stem cell biology. In addition retroviral transduction can be applied to organoids derived from pre-established transgenic mice as well as human patient samples. As knock-in and knock-out approaches cannot be extended to humans, the human SI organoids constitute an attractive alternative.

In summary, gene manipulation through retroviral transduction allows phenotypic analysis in small intestinal organoids derived from mice or human tissue samples, thereby complementing mouse genetics and cell lines while opening new avenues for studies in human derived tissues. Retroviral transduction enables gain- and loss-of-function studies to be performed in the organoid culture system^4^. This makes it a valuable resource for investigating gene function, adult stem cell biology and disease while being in accordance with the three Rs (reduction, refinement, and replacement).

## Protocol

All mice used in the following protocol were kept in specific pathogen-free conditions, and all procedures were performed according to the United Kingdom Home Office regulations.

### 1. Preparation

1. Prepare media 1 hr in advance of use (see **Table 1** and List of Materials for more details) and pre-warm in a 37 °C water bath at least 10 min before use.

### 2. Culturing Small Intestinal (SI) Organoids

NOTE: Unless otherwise stated, all incubations are performed at 37 °C, 5% CO_2_ in a humidified incubator. Equipment and reagents coming into contact with living cells must be sterile.

Pre-warming the tissue culture plate is important as it prevents the basement matrix (Matrigel or BME) drops from spreading out when seeding. Also, the basement matrix should be kept on ice at all times. Store at -20 °C and thaw on ice before use.

1. Isolating Crypts
  1. For isolation of the small intestine sacrifice the mouse according to the national rules and regulations, then place the animal on its back and wash the abdomen with 70% ethanol. Perform a longitudinal midline incision from the groin to the sternum. First, cut the skin and then the subcutaneous tissue. Remove the small intestine from the cecum to the stomach. Crypts isolated from duodenum, jejunum and ileum can be used for organoid culture.
  2. Wash the isolated small intestine with pre-cooled Phosphate-Buffered Saline without calcium or magnesium (PBS0).
  3. Cut the tissue into 3-5 cm long pieces and use scissors to cut it open longitudinally. Spread the tissue by using forceps.
  4. Scrape off villi using a coverslip. Caution, too much force will cause the tissue to tear and will reduce the yield of crypts in following steps.
  5. Transfer the tissue to a 50 ml tube containing pre-cooled PBS0 using forceps. Wash the tissue fragments through vigorous shaking and change the PBS0. Repeat 2-3x or until the PBS0 turns less cloudy.
  6. Incubate the tissue in a 50 ml tube containing 30 ml of PBS0 with 1 mM ethylenediaminetetraacetic acid (EDTA) for 30 min at 4 °C on a tube roller.
  7. Vigorously shake the tube and transfer the tissue to another 50 ml tube containing 30 ml of PBS0 with 5 mM EDTA. The 1 mM EDTA solution (previously containing the tissue) will contain a mixture of crypts and villi. This fraction is not suitable for seeding of organoids as it often contains a high percentage of villi.
  8. Incubate the tissue further for 1 hr at 4 °C on a tube roller.
  9. Vigorously shake the tube and collect the solution in a clean 50 ml tube. Confirm the presence of crypts and estimate the number, *e.g.*, by counting crypts in 50 µl by light microscopy. Calculate the volume needed to obtain 50-100 crypts and transfer it to a 1.5 ml tube.
  10. Spin at 300 x g for 5 min. Discard the supernatant, resuspend the pellet in 50 µl of basement matrix, and seed in a pre-warmed 24-well plate. Incubate the plate in a tissue culture incubator for 5-15 min so the basement matrix polymerizes.
  11. Overlay with 500 µl ENR medium (see **Table 1**). Approximately 24 hr after seeding the organoids will show a small round cystic shape and after another 2-3 days budding structures become visible. Change to fresh ENR media every 3 days.
  12. Passage organoid cultures every 7 days.
2. Passaging and Maintaining Organoids
  1. Maintain the organoids by refreshing the media every 3 days and passage 1:3 or 1:5 as the lumen becomes filled with dead cells, approximately every 7 days.
  2. Break the basement matrix dome with medium using 1 ml pipetman tip and transfer it from the well to a 1.5 ml tube.
  3. Mechanically dissociate the organoids through pipetting approximately 50x using a fine (*e.g.*, 200 µl) tip.
  4. Spin at 300 x g for 5 min.
  5. Discard the supernatant and resuspend the pellet in 150-250 µl of basement matrix. Seed in a pre-warmed 24-well plate (50 µl of basement matrix /well). Before seeding pipette the basement matrix up and down once to coat the wall of the tip. Pipette slowly to avoid bubbles and seed in the middle of the well to prevent the basement matrix from spreading out. It is desired to get a dome of basement matrix in the center of the well.
  6. Incubate in a tissue culture incubator for 5-15 min so the basement matrix polymerizes. Overlay with 500 µl ENR media per well.

### 3. Pre-infection Treatment of SI Organoids

NOTE: **Figure 1** illustrates the transduction procedure.

1. Exchange ENR to ENRWntNic (see **Table 1**) medium and grow the organoids in this medium for a minimum of 3 days or until they have adopted a cystic morphology. Wnt3a increases the number of stem and Paneth cells while Nicotinamide (Nic) improves culture efficiency.

### 4. Virus Production

1. One 150 mm dish of Platinum-E cells is needed per infection. Seed approximately 5 x 10^6^ cells with 15-18 ml media (DMEM + 10% FBS) in the presence of puromycin (1 µg/ml) and blasticidin (10 µg/ml). Transfect cells after 2-3 days, when they have reached 70-80% confluency. Change the medium to DMEM + 10% FBS without puromycin and blasticidin prior to transfection.
2. Add 30 µg of retroviral DNA construct and 240 µl of polyethylenimine (PEI) to separate tubes containing 1 ml of opti-MEM. Mix and incubate at room temperature for 5 min.
3. Pool the two solutions and incubate at room temperature for 20-30 min. Add the complete mixture to the medium of Platinum-E cells and carefully shake the plate to ensure equal distribution of the DNA-PEI complexes.
4. Incubate the Platinum-E cells with the transfection mixture overnight and refresh the medium the following day. Keep the cells in the new medium for 2 days.
5. Collect only the medium in a 50 ml Falcon tube, pass it through a 0.45 µm filter and centrifuge at 8,000 x g at 4 °C for 12-16 hr. Discard the supernatant and resuspend the pellet in 250 µl of Transduction medium (see **Table 1**).

### 5. Organoid Fragment Preparation

1. For one infection one well from a 24-well plate is needed. Break the basement matrix dome with medium using 1 ml pipetman tip and transfer it to a 1.5 ml tube.
2. Use a finer volume tip (*e.g.*, 200 µl tips) to mechanically disrupt the organoids through pipetting (30-50x). The solution will become cloudy and no whole organoids should be visible.
3. Centrifuge at room temperature, 900 x g for 5 min.
4. Discard the supernatant and resuspend the pellet in 500 µl of cell culture grade recombinant protease (*e.g.*, TrypLE). Incubate at 37 °C for 5 min. Following incubation check the size of the organoid fragments using light microscopy, counting the number of cells per fragment. Fragments containing 5-10 cells are ideal. If the majority of the fragments contain a higher number of cells the incubation time can be increased by 2 min at a time.
5. Terminate the dissociation process by adding 500 µl of ENR medium.
6. Centrifuge at room temperature, 900 x g for 5 min. Remove the supernatant and keep the pellet on ice or 4 °C.

### 6. Retroviral Transduction

1. Combine organoid fragments with 250 µl of the retroviral solution (from section 4.5) in one well of a 48-well plate. Mix gently by slow pipetting using 1 ml pipetman tip.
2. Seal the plate with Parafilm.

### 7. Spinoculation and Plating

1. Centrifuge the plate at 32 °C, 600 x g, for 1 hr. Carefully remove the Parafilm and incubate the plate in a tissue culture incubator for 6 hr.

### 8. Seeding of Infected Organoid Fragments

1. Transfer the infected organoid fragments and transduction media from the well to a 1.5 ml tube, and spin at 900 x g for 5 min.
2. Discard the supernatant and put the tube containing the pellet on ice for 5 min to cool. Add 100 µl of basement matrix and resuspend the pellet by pipetting slowly up and down.
3. Seed drops of 50 µl ‘basement matrix -cell mix’ in a new 24-well plate. Incubate the plate at 37 °C for 5-15 min until the basement matrix solidifies.
4. Add transduction media without polybrene to the wells and incubate the plate in a tissue culture incubator. Change media every 2-3 days.

### 9. Selection

1. Start selection after 2-3 days by adding puromycin (1 µg/ml) to the media.
2. When the fragments are starting to form organoids replace the Transduction media with ENR media supplemented with puromycin (1 µg/ml).

### 10. Post-infection Treatment of SI Organoids

1. Culture transduced organoids according to the “Passaging and maintaining organoids” protocol (section 2.2.1). After 1-2 weeks the SI organoids will regain their budding structures.
2. Following the appearance of budding structures induce the expression of miRNA or cDNA by adding 4-hydroxytamoxifen (4-OHT) in a working concentration of 1 µM. Note that this step is valid only if using retroviral vectors from Addgene (pMSCV-loxp-dsRed-loxp-eGFP-Puro-WPRE (32702), pMSCV-loxp-dsRed-loxp-3xHA-Puro-WPRE (32703), and pMSCV-FLIP-puro-dsRed-GFP-miRNA (32704)) and organoids expressing Cre-ERT2.

### 11. Confirmation of Infection and Expression/Suppression of the Gene of Interest

1. If using retroviral vectors from Addgene (32702, 32703 and 32704) transduction efficiency can be confirmed by observation of dsRed expression. Furthermore, the expression or suppression of the gene of interest can be confirmed by Western Blot, using the GFP or 3xHA epitope (for gene overexpression) and qPCR (for gene knockdown), as exemplified in Koo *et al*^4^.

## Representative Results

Organoids are ready to be split when the central lumen is darkened due to the presence of dead cells (**Figure 2**). After 2-3 days of pre-treatment organoids should adopt a round cystic morphology (**Figure 3**). This increases the number of stem cells, enhancing the chances of obtaining stable integration. The size of the viral pellet may vary following centrifugation of the viral supernatant, most likely due to varying contribution of cell debris to the pellet size. No clear correlation to transduction efficiency has been observed. During the selection procedure non-transduced organoids will die, while the ones having a stable integration will remain. The fluorescent protein from MSCV-eGFP retrovirus can be observed in cells originating from surviving organoids, which have a cystic morphology, within 2-3 days following transduction (**Figure 4**).


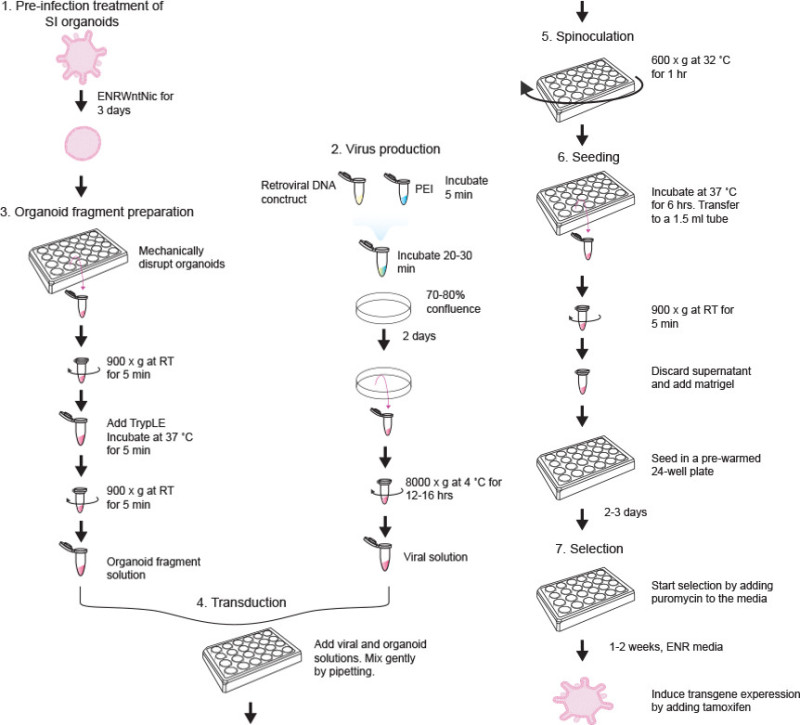
**Figure 1. A schematic drawing of the retroviral transduction procedure.** Prior to infection organoids are pre-treated using ENRWntNic until they adopt a cystic structure (step 1). Platinum-E cells are used as the packaging cell line, and are cultured until they reach 70-80% confluency. Thereafter they are transfected with the retroviral construct using PEI. Viruses are harvested 2 days later (step 2). Organoids are trypsinized to obtain fragments containing 1-10 cells (step 3), and are then infected (step 4). Following spinoculation to increase the infection efficiency (step 5), the infected organoid fragments are seeded (step 6) and 2-3 days later selection for positive clones with stable integration can be performed (step 7).


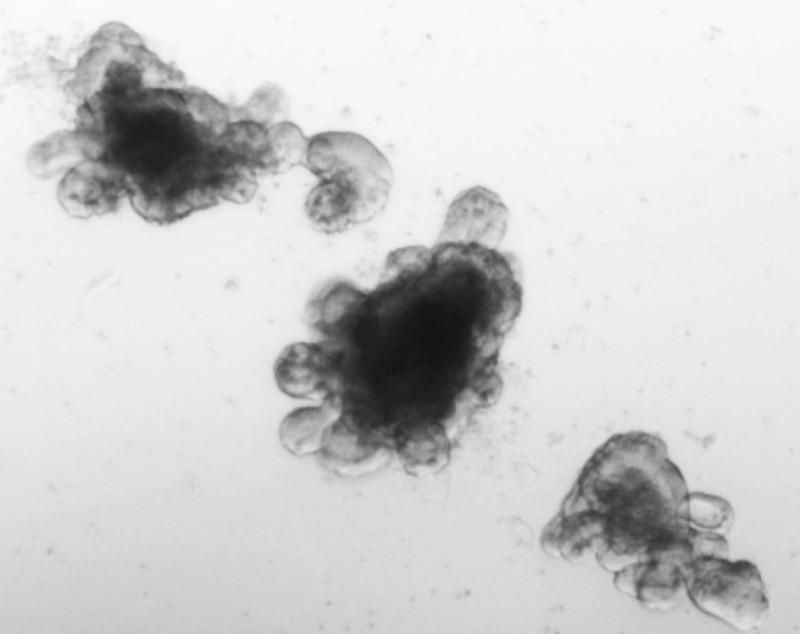
**Figure 2. Representative image of organoids after 4-6 days of culture.** The lumen is filled with dead cells, making it appear dark. Organoids at this stage are ready to be passaged.


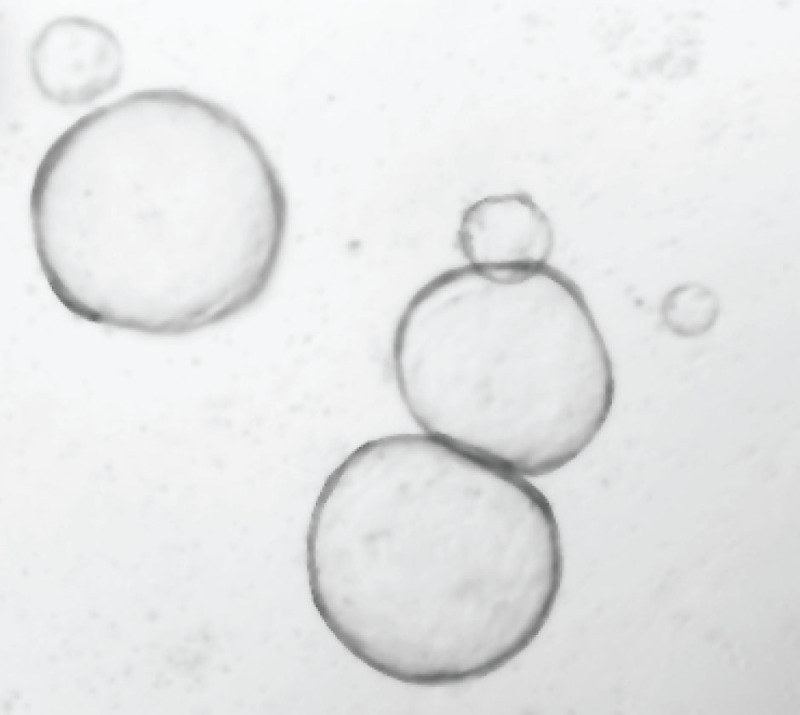
**Figure 3. Representative image of small intestinal organoids cultured in ENRWntNic media for 3-4 days.** The organoids adopt a cystic morphology.


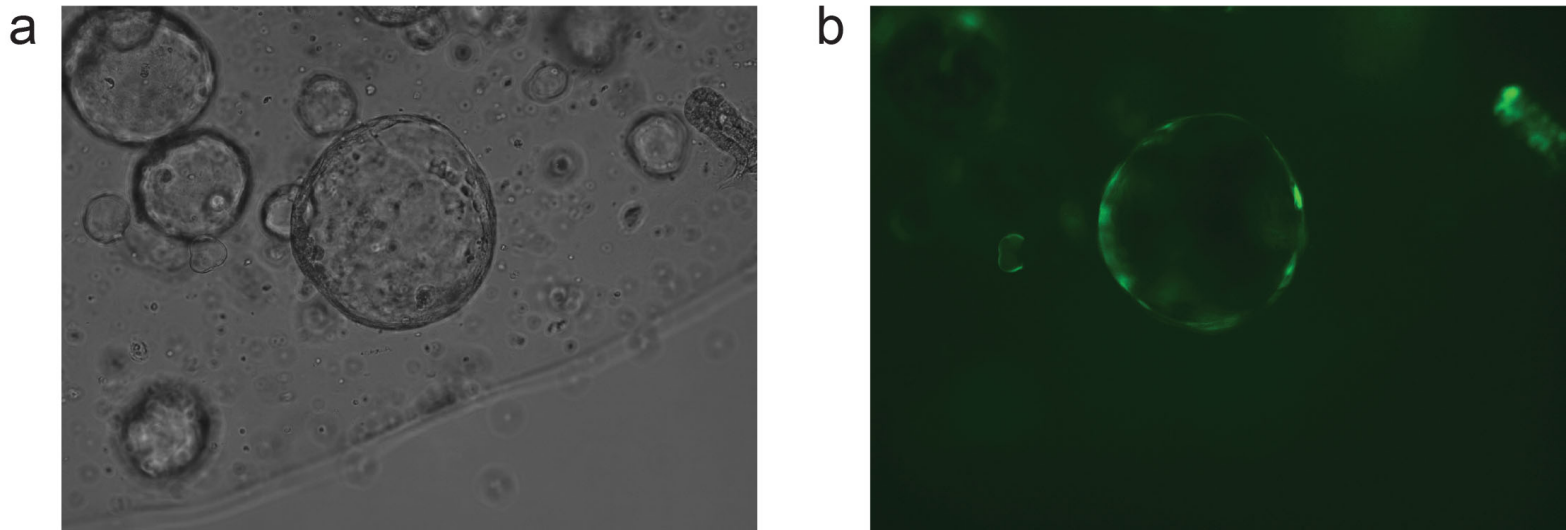
 **Figure 4. Representative image of small intestinal organoids (a) that show viral transgene expression (b, eGFP).**

**Table:** 

**Advanced DMEM/F12 +++**
Store at 4 °C for 4 weeks
Advanced DMEM/F12	500 ml
Glutamax 100x	5 ml
Hepes 1 M	5 ml
Antibiotics 100x	5 ml
**ENRWntNic medium (for 20 ml)**
Store at 4 °C for 2 weeks
Advanced DMEM/F12 +++	7.2 ml
B27 supplement (50x)	400 µl
N2 supplement (100x)	200 µl
n-Acetylcysteine (500 mM)	50 µl
mouse EGF (500 µg/ml)	2 µl
mouse Noggin (100 µg/ml)	20 µl
R-Spondin conditioned medium	2 ml
Wnt3a conditioned medium	10 ml
Nicotinamide (1 M)	200 µl
**Transduction medium (for 20 ml)**
Prepare fresh
ENRWntNic medium	20 ml
Y-27632 (10 µM)	20 µl
Polybrene (8 µg/ml)	20 µl
**ENR medium (for 20 ml)**
Store at 4 °C for 4 weeks
Advanced DMEM/F12 +++	17.4 ml
B27 supplement (50x)	400 µl
N2 supplement (100x)	200 µl
n-Acetylcysteine (500 mM)	50 µl
mouse EGF (500 µg/ml)	2 µl
mouse Noggin (100 µg/ml)	20 µl
R-Spondin conditioned medium	2 ml
**Media for Platinum-E cells (for 500 ml)**
Store at 4 °C for 12 weeks
DMEM	449.45 ml
Fetal Bovine Serum (FBS)	50 ml
Puromycin (1 μg/ml)	50 µl
Blasticidin (10 μg/ml)	500 µl

**Table 1. Media composition for Advanced DMEM/F12 +++, ENRWntNic medium, Transduction medium, ENR medium, and medium for Platinum-E cells.**

## Discussion

To achieve high transduction efficiency certain aspects are critical. One is the pre-treatment of organoids with ENRWntNic media until they adopt a round cystic shape. This increases the number of stem cells and thereby the chance of obtaining a stable integration of the transgene, as well as increasing the survival rate of the SI organoids throughout the transduction procedure. Another parameter is the incubation time following spinoculation. Too short or too long incubation results in poor transduction efficiency and poor survival of the organoids, respectively. The spinoculation step is not essential although it significantly increases the percentage of transduced organoids. Finally, high-titer virus is key for successful transduction. This is dependent on the type of packaging cell line and virus. The combination of Platinum-E cell line and murine stem cell virus (MSCV), was found to produce a titer high enough for transduction of organoids.

Below are tips for troubleshooting which may help to achieve successful transduction. First, if the transfection of the packaging cell line is poor, make sure that the confluency of the cells is between 70-80% and that the incubation time of the pooled PEI-DNA mixture is between 20-30 min. The survival of the organoids during transduction highly depends on the fragment size. Too long trypsinization causes the majority of fragments to consist of less than 3 cells and thereby decreases organoid survivability. Another factor is the activity of the Wnt conditioned medium, if the activity is too low boosting it through addition of CHIR99021 in a working concentration of 5 μM can increase the survival. CHIR99021 inhibits GSK3, resulting in increased Wnt signaling. Furthermore, Y-27362, which prevents anoikis is added to the transduction media to improve organoid survivability, since the organoids are disrupted to fragments (containing 1-10 cells) prior to transduction. As mentioned above, the incubation time after spinoculation should not exceed 6 hr. Lastly, if poor transduction is observed the aforementioned factors influencing the viral titer and the size limit of the insert for the retroviral vector should be considered. The efficiency of the knockdown is highly dependent on the miRNA. Since the efficiency varies with the combination of the target gene and miRNA it is worth performing an efficiency screen to identify those that work best.

The technique is restricted to the epithelial phenomena of the organoid system. In the future it might be possible to study infectious or immune-mediated diseases through co-culture of pathogens or reconstitution with components derived from the immune system, respectively. Moreover, retroviruses can only carry inserts of a relatively small size. Consequently, naturally occurring regulatory regions have to be excluded and therefore the expression of the transgene cannot mimic that of the endogenous gene. As mentioned above, the knockdown efficiency is dependent on the target gene and miRNA. If no miRNA with suitable knockdown efficiency can be found it may limit the use of the technique for that particular target gene.

Theoretically, organoids are compatible with all standardized manipulative techniques used for cell lines. Retroviral transduction was the first method to be reported^4^, and recently BAC (bacterial artificial chromosome)-transgenesis has become available^5^. With a total generation time of 2-3 weeks, after transfection of the viral plasmid into the packaging cell line, it is significantly faster than the generation of a transgenic (tg) mouse. By maintaining the *in vivo *crypt-villus architecture while containing stem cells as well as all differentiated cell lineages of the intestinal epithelium, the organoid culture system bridges the gap between tg animal and previously used cell culture.

The protocol described here provides a method to perform phenotypic analysis of endodermal epithelium* in vitro* through gain- and loss- of function studies. This makes it feasible to address physiologically relevant questions in adult stem cell biology, with a minimal need of tg mice. For example, the generation of conditional knockout mice could be avoided by using organoids derived from newborn mutants with perinatal lethality^6^. In addition, the technique can be applied to organoids derived from previously established knockout mice to study the role of paralogues by performing additional knockdown^7,8^.

Following the establishment of small intestinal organoids, adaptation of the original culture protocol has allowed culturing of pancreatic, liver, colon and stomach epithelia^9-11^. Furthermore, human intestinal organoids and tumor organoids have been derived from normal human biopsies, primary adenoma and colorectal cancer biopsies^10^. The viral infection protocol can easily be extended to these types of organoids and provides an unprecedented way of performing functional studies in human derived tissues.

Taken together, retroviral transduction of small intestinal organoids is a valuable resource for investigating stem cell maintenance, differentiation, and cell fate decision, as well as cell signaling and cell- cell interactions.

## Disclosures

The authors have nothing to disclose. The authors have no conflict of interest declared.
